# Liquid Chromatography with Electrospray Ionization and Tandem Mass Spectrometry Applied in the Quantitative Analysis of Chitin-Derived Glucosamine for a Rapid Estimation of Fungal Biomass in Soil

**DOI:** 10.1155/2016/9269357

**Published:** 2016-02-09

**Authors:** Madelen A. Olofsson, Dan Bylund

**Affiliations:** Department of Natural Sciences, Mid Sweden University, 851 70 Sundsvall, Sweden

## Abstract

This method employs liquid chromatography-tandem mass spectrometry to rapidly quantify chitin-derived glucosamine for estimating fungal biomass. Analyte retention was achieved using hydrophilic interaction liquid chromatography, with a zwitter-ionic stationary phase (ZIC-HILIC), and isocratic elution using 60% 5 mM ammonium formate buffer (pH 3.0) and 40% ACN. Inclusion of muramic acid and its chromatographic separation from glucosamine enabled calculation of the bacterial contribution to the latter. Galactosamine, an isobaric isomer to glucosamine, found in significant amounts in soil samples, was also investigated. The two isomers form the same precursor and product ions and could not be chromatographically separated using this rapid method. Instead, glucosamine and galactosamine were distinguished mathematically, using the linear relationships describing the differences in product ion intensities for the two analytes. The* m/z* transitions of 180 → 72 and 180 → 84 were applied for the detection of glucosamine and galactosamine and that of 252 → 126 for muramic acid. Limits of detection were in the nanomolar range for all included analytes. The total analysis time was 6 min, providing a high sample throughput method.

## 1. Introduction

Estimation of fungal biomass is useful in the study of nutrient and energy flow relationships in soil and when evaluating fungal infection in food and plant material. Various approaches are available to achieve these ends [1]. Direct quantification through histological analysis [2] is one approach, although its tediousness and tendency to deliver biased values are known disadvantages [3]. Indirect quantification using biochemical markers, on the other hand, can deliver more standardized results. Ergosterol, a sterol found almost exclusively in fungal membranes, can be extracted with MeOH or EtOH and quantified using liquid chromatography-ultraviolet (LC-UV) detection [4, 5]. As ergosterol is believed to be relatively unstable and subject to degradation after fungal death, its quantification is generally assumed to estimate the biomass of metabolically active fungi [4, 6]. Quantification of phospholipid-derived fatty acids (PLFAs) using gas chromatography is also an alternative [7]. Phospholipids constitute the main structural elements in the cellular membranes of all living organisms with the exception of archaea, and as PLFAs vary structurally with source they provide more or less selective biomarkers for different species of fungi as well as bacteria [8].

Chitin, a naturally occurring polymer of* N*-acetyl-D-glucosamine, is the structural building block of fungal cell walls. As invertebrate exoskeletons also contain this polymer, microarthropods can contribute to chitin content in soil, albeit in conceivably minute amounts as arthropod soil biomass is the minimal compared with that of soil microorganisms [9]. Chitin is considered more resistant to degradation than ergosterol and phospholipids and is believed to have a recalcitrant portion of 10 to 15% of the original biomass [10]. Amino sugar-containing polymers are considered to contribute to 5 to 12% of soil organic N [11].

Through acid hydrolysis chitin can be degraded into the amino sugar glucosamine (GlcN) [12, 13], which in turn can be quantified to estimate total fungal biomass. An alternative is to treat chitin with an alkaline solution to form chitosan, an* N*-deacetylated and partly depolymerized product [14]. Another source of GlcN in soil is peptidoglycan, from bacterial cell walls. Peptidoglycan also contains muramic acid (MA), which is exclusively found in bacteria [15] normally at a 1 : 1 ratio with GlcN [16], though this may vary in some bacterial species [17]. GlcN is also found to some extent in higher plants, typically as glycoproteins in seeds and in fungal and bacteria-produced antibiotics [15].

Various colorimetric assays have been used in the quantification of GlcN and chitosan. Elson and coworkers [18, 19] used* p*-dimethylaminobenzaldehyde (Ehrlich reagent) to form a red-colored product with both GlcN and chitosan. Later, Tsuji and coworkers [20, 21] developed a procedure, also used by Ride and Drysdale [14], where deaminated* N*-acetylglucosamine was treated with 3-methyl-2-benzothiazolone hydrazone hydrochloride (MBTH) and ferric chloride to yield an intense blue color which could be measured spectrophotometrically.

Many amino sugars are associated with microorganisms, though GlcN, galactosamine (GalN), mannosamine (ManN), and MA are the ones most commonly found in soil [22]. Chromatographic separation of amino sugars can be performed in gas or liquid phases. Separation with gas chromatography (GC) is possible following analyte derivatization and subsequent flame ionization detection [23, 24]. Reversed-phase (RP) LC-fluorescence detection is also applicable for the quantification of GlcN and other amino sugars. In such applications, the analytes are derivatized with a fluorescent reagent, for example, 9-fluorenylmethyl chloroformate (FMOC-Cl) or* o*-phthalaldehyde (OPA), either manually [12, 25] or automatically using precolumn derivatization [26, 27]. Derivatization not only enables florescence detection, but also improves retention on the otherwise excessively hydrophobic stationary phases of RP columns. An alternative to RP separation is anion-exchange chromatography with pulsed amperometric detection [28]. Advanced analysis of fungal turnover, measuring the ratio of ^13^C to ^12^C for amino sugars, is possible via liquid chromatography-isotope ratio mass spectrometry [29, 30] or GC-combustion-isotope ratio mass spectrometry [31]. Quantitative analysis of underivatized GlcN in blood plasma and synovial fluid has lately been realized in the pharmaceutical field via HILIC separation and mass spectrometry (MS) detection [32–34].

**Figure 1:**
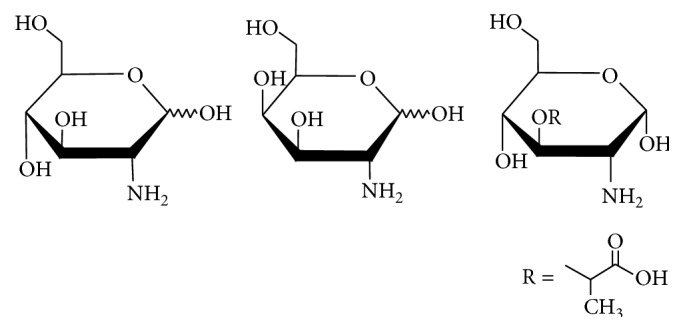
Chemical structures of (from left to right) glucosamine, galactosamine, and muramic acid.

This project aimed to develop a simple and rapid method for the estimation of fungal biomass by quantification of underivatized, chitin-derived GlcN in soil, applying LC separation with electrospray ionization and tandem mass spectrometry detection (ESI-MS/MS). MA was included in the analytical method to enable calculation of the bacterial contribution to the total GlcN concentration. In addition, the possibility to distinguish GalN from GlcN by comparing product ion intensities is presented. Chemical structures of GlcN, GalN, and MA can be seen in Figure 1.

## 2. Material and Method

### 2.1. Chemicals and Equipment

10 mM solutions of D-glucosamine hydrochloride (Alfa Aesar, Karlsruhe, Germany) and D-galactosamine hydrochloride (Sigma-Aldrich, St. Louise, USA) were prepared in ultrapure water (MilliQ, Millipore, Bedford, MA). Muramic acid (Sigma-Aldrich, St. Louise, USA) standard solution was prepared by dissolving 5 mg in 10 mL ultrapure water resulting in an approximate analyte concentration of 2 mM. In addition, a 10 mM solution of D-mannosamine hydrochloride (Sigma-Aldrich, St. Louise, USA) was prepared. The standard solutions were stored at −20°C until diluted into working standards. Formic acid for mass spectrometry (Sigma-Aldrich, St. Louis, MO), ammonia (Merck, Darmstadt, Germany), and ACN (for HPLC) from VWR were used in eluents.

The instrumental setup included the following: a Shimadzu LC-10AD isocratic pump (Kyoto, Japan), a mobile phase degassing unit (Uniflows, Tokyo, Japan), an Agilent 1100 autoinjector with appurtenant thermostat (Santa Clara, CA) where the samples were held at 4°C prior injection, a ZIC-HILIC analytical column (150 × 2.1 mm, 5 *μ*m) from SeQuant (Umeå, Sweden), and an API3000 mass spectrometer (AB Sciex, Concord, Canada).

### 2.2. LC-ESI-MS/MS Analysis

To achieve sufficient retention and acceptable peak shape, isocratic elution was investigated by combining 5 mM ammonium formate buffer (pH 3.0) and ACN at different percentages. Electrospray and mass spectrometry parameters including collision induced dissociation (CID) analyte fragmentation were investigated via direct infusion of reference material dissolved in the determined mobile phase composition, using a syringe pump (Harvard Apparatus, Holliston, MA). Gas flow settings in the ESI interface and collision cell were optimized manually while analyte-specific potential settings and collision energies were optimized from ramping experiments.

### 2.3. Method Validation

Limit of detection (LOD) was defined as the injected concentration resulting in a peak height three times the baseline noise level and limit of quantification (LOQ) as the injected concentration resulting in a peak height ten times the baseline noise level. LOD and LOQ were examined through repeated injections of reference material. Linearity was investigated from concentrations corresponding to individual LOQ values up to 40 *μ*M. Precision specific to the peak area was determined via repeated injections of 1 and 10 *μ*M standard solution and expressed as relative standard deviation (RSD%). All standard solutions were dissolved in 1 : 1 ACN : ultrapure water.

A number of soil samples were treated according to Ekblad and Nasholm [12], with some minor adjustments. In short, dried and grounded soil was treated with 0.2 N NaOH at 70°C for 17 h, washed four times with ultrapure water, dried, and finally subjected to acid hydrolysis by additions of 6 M HCl and sample incubation at 70°C for 16 h. Prior to analysis, the acid hydrolysate was filtered through Whatman 0.45 *μ*m membrane filters (Clifton, NJ), evaporated in N_2_ flow, and finally resolved in 1 : 1 ACN : ultrapure water. These samples were analyzed in duplicate and in random order to assess method applicability. Withal, reference material was added to sample matrix to investigate analyte recovery. Standard additions were made at two levels, low and high, corresponding to final sample concentrations of 5 and 20 *μ*M, respectively. Sample matrix without standard addition, but with the equivalent additions of 1 : 1 ACN : ultrapure water, was also analyzed. Six replicates of low, high, and zero additions were analyzed.

## 3. Results and Discussion

### 3.1. Analytical Parameters

This study aimed to find a simple, rapid method for quantifying chitin-derived GlcN without the need for analyte derivatization. Current methods for quantification of amino sugars in soil use derivatization, which necessitates further sample preparation and additional use of chemicals. Automatic derivatization requires specialized autoinjectors, and manual derivatization can introduce human error. Derivatization is not necessary in MS detection as the analytes are detected according to mass to charge ratio (*m/z*). MS/MS detection applying multiple reaction monitoring (MRM) mode is also highly selective, which is favorable when analyzing complex sample matrices.

Since underivatized amino sugars are too polar to be retained on RP stationary phases, HILIC was tested for this purpose. HILIC combines hydrophilic stationary phases with typical RP mobile phases, enabling the retention of polar and hydrophilic analytes [35]. Standard HILIC mobile phases are also compatible with MS detection and typically consist of 40–97% of ACN or other water-miscible organic solvents. The higher the organic solvent percentage, the higher the retention of analytes. For this application, a zwitter-ionic stationary phase was chosen, consisting of positively charged quaternary ammonium and negatively charged sulfonate groups (1 : 1) able to retain both positively and negatively charges analytes.

### 3.2. Amino Sugar Isomers

GlcN, GalN, and ManN are isobaric amino sugars found in soil. They all produce identical precursor ions in terms of* m/z* when analyzed with MS. They also give rise to the same product ions when subjected to CID. These are therefore undistinguishable merely using MS or MS/MS detection. Even though GlcN is the analyte of interest for the indirect estimation of fungal biomass, coquantification with its isomers may lead to inaccurately high values. For this reason, chromatographic separation of the three isobaric amino sugars was initially tested using gradient elution from a high to low ACN percentage. These attempts proved futile, and even the *α*- and *β*-anomers of the amino sugars appeared to separate more readily than the isomers themselves.

**Figure 2:**
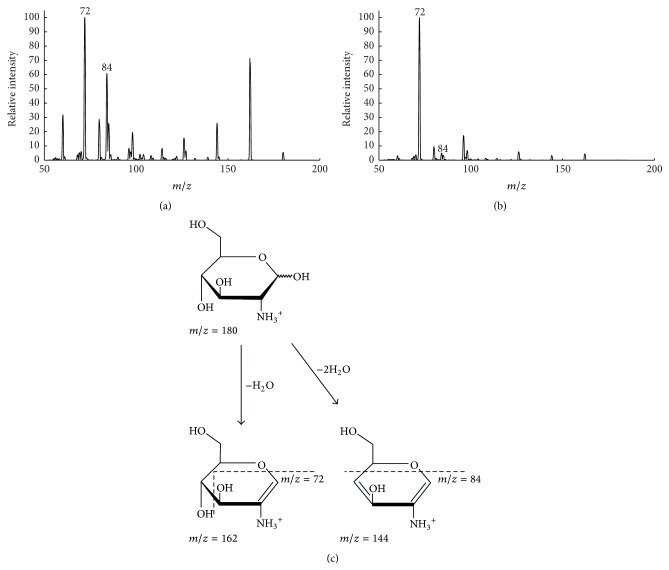
Positive ESI-MS/MS product ion spectra of (a) glucosamine and (b) galactosamine, produced through direct infusion of 100 *μ*M reference solutions of each analyte with a collision energy of 25 V. Suggested fragmentation of glucosamine (c) presenting the formation of the two* m/z* transitions investigated in the method (180 → 72 and 180 → 84). Fragmentation of galactosamine is believed to follow the same route but with less formation of the 84 fragment due to the conformation of the C4 hydroxy group.

The most intense product ion produced by GlcN, GalN, and ManN had an* m/z* value of 72, making it a natural choice for quantification purposes. A closer investigation of the fragmentation patterns of GlcN and GalN, however, revealed differences in intensity among the additional fragments (Figure 2). The most striking difference was observed for the fragment with the* m/z* value of 84 Th, which is produced in significantly greater proportion for GlcN than for GalN. Presumably, these differences in fragment intensities are due to the difference in conformation of the hydroxy group at C4 and consequently the readiness to lose a second H_2_O group (Figure 2(c)). These differences were not observed between GlcN and ManN, where nearly identical product ion spectra were produced. Since the contribution of ManN to the total amino sugar content in soil is reported to be relatively low, up to 50 times less than GlcN in grass land [36], its distinction was not considered to be critical in quantifying chitin-derived GlcN, and it is therefore not considered further.

The difference found in fragment intensity between GlcN and GalN suggested that they could be distinguished mathematically. Initial tests showed that the ratios between the 72 and 84 Th fragments were reproducible at different analyte concentrations. A full factorial design using Minitab 16 (Minitab Inc., State Collage, Pennsylvania) was performed to investigate this further. Combinations of three levels of individual amino sugar concentrations in mobile phase were analyzed in random order and with two replicates. The design can be found in Table 1. GalN was investigated at higher concentrations compared to GlcN, due to its low formation of the 84 Th fragment.

The results from the full factorial design are also reported in Table 1. A regression analysis was performed on the given areas to investigate a potential relationship. The following linear relationships were found:

(1) A72=k1×CGlcN+k2×CGalN,

(2) A84=k3×CGlcN+k4×CGalN.

*A*_72_ and *A*_84_ represent areas of the 72 and 84 Th product ions, respectively, and *k*_1–4_ represents the coefficients of slope specific for each combination of product ion and analyte (GlcN and GalN). The multiple linear regression models were significant with high *R* ^2^ values (97.6 and 98.2% for the 72 and 84 Th fragments, resp.), and no significant lack-of-fit was noted; that is, the models are valid over the entire range of concentrations studied.

When solving for *C*_GlcN_ and *C*_GalN_ using (1) and (2), the following equations were derived:

(3) CGlcN=A72−k2×CGalNk1,

(4) CGalN=A84−k3×A72/k1k4−k2×k3/k1.

With (3) and (4), corresponding concentration values for GlcN and GalN can be calculated. This was verified by inserting coefficients of slope from calibration curves determined from standard solutions consisting of reference material in mobile phase, analyzed within the same acquisition batch as the solutions representing the full factorial design. The calculated concentrations are also presented in Table 1 and were found to be on average 10 and 2% above the true values for GlcN and GalN, respectively.

### 3.3. Final LC-ESI-MS/MS Method

**Figure 3:**
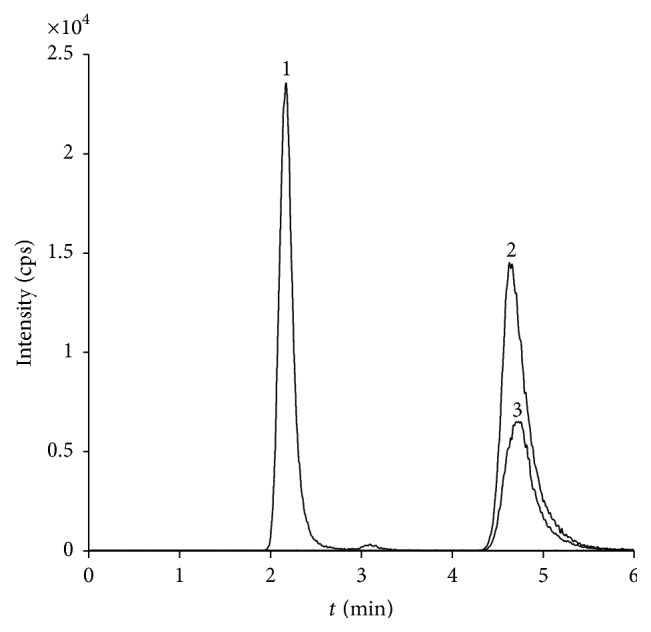
MRM chromatogram of a 10 *μ*M standard solution of (1) muramic acid and (2) glucosamine (180 > 72) and (3) glucosamine (180 > 84) separated on a ZIC-HILIC column (150 × 2.1 mm, 5 *μ*m).

The sample injection volume was 4 *μ*L and the syringe was washed between injections to prevent carry-over effects. Isocratic elution was then achieved using a mobile phase consisting of 60% 5 mM ammonium formate buffer (pH 3.0) and 40% ACN, delivered at a flow rate of 0.3 mL/min. Before entering the ESI interface, the total flow was split using a Valco tee union resulting in a continuous flow of approximately 60 *μ*L/min, which is more suitable for ESI. MRM was applied with an analyte* m/z* transition of 180 → 72 for GlcN, in accordance with Roda et al. [32]. The same optimal* m/z* transition was applied for GalN. MA was detected at* m/z* transition 252 → 126, in accordance with Black et al. [37]. In addition, the* m/z* transition of 180 → 84 was included for the mathematical distinction of GlcN and GalN. The amino sugars were analyzed in positive mode applying an ionization voltage of 4500 V. Optimal and analyte-specific declustering, focusing, and entrance potentials are reported in Table 2. Nebulizer and curtain gas settings were 10 and 8 arbitrary units of N_2_, respectively. Collision gas was set to 5 arbitrary units of N_2_ in order to achieve the most favorable fragmentation. Analyte-specific collision energies and collision cell exit potentials are also reported in Table 2. MA eluted at 2.1 min and GlcN and GalN at 4.5 min (Figure 3), and the total analysis time was 6 min.

### 3.4. Method Validation

Method validation was performed on all three analytes and for both the 72 and 84 Th fragments for GlcN and GalN. Linearity was found to be excellent from LOQ values up to 40 *μ*M. At higher concentrations, the response gradually leveled off. Precision at low and high concentrations lay between 0.6 and 1.8%, and LOD values are between 10 and 500 nM. A complete list of figures of merits for GlcN, GalN, and MA is presented in Table 3.

In the recovery trial, recoveries of 95 to 105% were obtained for MA. Somewhat lower recoveries, in the range of 75 to 85%, were obtained for GlcN and GalN irrespective of the* m/z* transition (180 > 72 or 180 > 84) investigated. In order to investigate the possibility of coeluting matrix interferences causing this suboptimal recovery, samples were analyzed in full scan mode using both positive and negative electrospray. Possible interferences were discovered, eluting just before GlcN/GalN at 4.2 min, with* m/z* values of 64 and 105. These values correspond with the masses of [ACN + Na]^+^ and [2ACN + Na]^+^. If correctly interpreted, this indicates that a large amount of Na^+^ is present in the sample, presumably as remnants from the NaOH treatment prior to acid hydrolysis in the sample extraction. It is possible that the repeated washing steps did not adequately remove the access Na^+^. The addition of NaOH is believed to remove proteins and amino acids, possible interfering with analysis [12]. This step is, however, often excluded [24, 26, 28–30], which would presumably result in fewer matrix-related problems when applying the analysis method reported here.

### 3.5. Method Application

**Figure 4:**
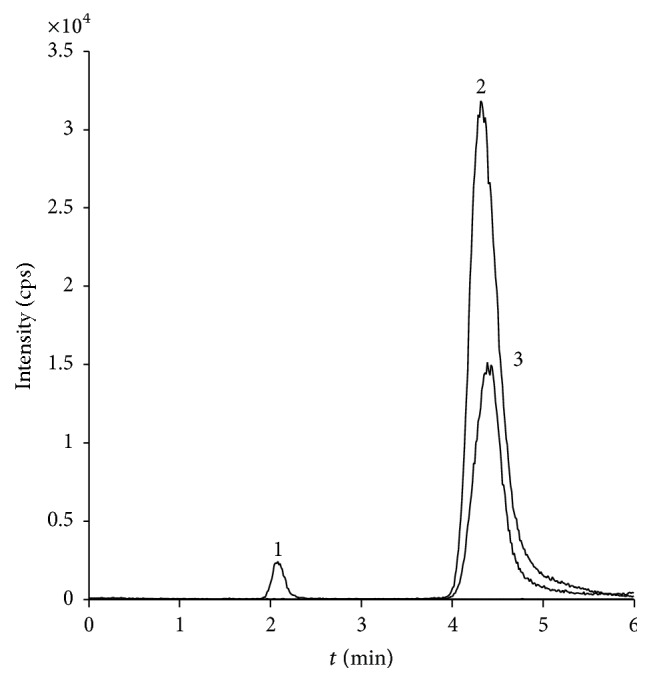
MRM chromatogram of an extracted soil sample with peaks representing* m/z* transitions (1) 252 → 126, (2) 180 → 72, and (3) 180 → 84. Peak areas correspond to 0.5 *μ*M of muramic acid and 15.3 and 1.4 *μ*M of glucosamine and galactosamine, respectively.

Nineteen soil samples, prepared according to the description in Section 2.3, were analyzed with duplicate injections in random order using the presented method. GlcN and GalN concentrations were calculated using (3) and (4) with slope coefficients (*k*) determined from reference solutions of GlcN and GalN, analyzed within the same acquisition batch. MA was also quantified for the samples, and derived concentrations for the three analytes were compared in terms of relative standard deviation for the duplicate samples. The pooled relative standard deviations for all nineteen samples were 4, 3, and 5% for MA, GlcN, and GalN, respectively.  Figure 4 represents one of the extracted soil samples. Calculated concentrations for GlcN and GalN were 15.3 and 1.4 *μ*M, respectively. MA concentration was 0.5 *μ*M, which can be subtracted from the calculated GlcN concentration to compensate for bacterial contribution.

## 4. Conclusions

This paper presents a simple, rapid approach for the estimation of fungal biomass via quantification of chitin-derived GlcN, using LC-ESI-MS/MS with isocratic elution. We believe that this method can beneficially complement the more complex and time-consuming methods that employ analyte derivatization to achieve acceptable separation of the four amino sugars discussed in this paper. By implementing MA in the analytical method, it is possible to deduce the bacterial contribution to the GlcN concentration. MA and GlcN are separated applying HILIC separation, and derivatization is not necessary to achieve chromatographic retention and enable detection. Additionally, a linear relationship was found for GlcN and its isomer GalN by investigating intensity differences for two* m/z* transitions (180 → 72 and 180 → 84), making it possible to mathematically calculate their respective contributions. The total analysis time is 6 min, allowing analysis of high sample throughput to be performed.

## Figures and Tables

**Table 1:** In and output values of the full factorial design used to investigate the linear relationship of peak area for the 72 and 84 Th fragments in relation to known concentrations of glucosamine (GlcN) and galactosamine (GalN). Experiments were analyzed in random order.

	Conc.^1^ (*μ*M)		Peak area (cps)		Calculated conc.^2^ (*μ*M)	
Experiment	GlcN	GalN	180 > 72	180 > 84	GlcN	GalN
1	1	5	3.6*E*5	3.3*E*4	1	5
2	1	10	5.8*E*5	4.1*E*4	1	10
3	1	15	9.9*E*5	6.1*E*4	1	16
4	8	5	5.3*E*5	1.3*E*5	9	5
5	8	10	8.0*E*5	1.4*E*5	9	10
6	8	15	1.2*E*6	1.7*E*5	9	16
7	15	5	6.9*E*5	2.2*E*5	16	5
8	15	10	9.7*E*5	2.3*E*5	16	10
9	15	15	1.4*E*6	2.8*E*5	16	15
10	1	5	3.3*E*5	2.9*E*4	1	5
11	1	10	6.0*E*5	4.1*E*4	1	11
12	1	15	1.0*E*6	6.1*E*4	1	16
13	8	5	5.9*E*5	1.5*E*5	9	5
14	8	10	9.0*E*5	1.6*E*5	9	11
15	8	15	1.2*E*6	1.7*E*5	8	15
16	15	5	7.9*E*5	2.6*E*5	16	5
17	15	10	9.8*E*5	2.4*E*5	17	10
18	15	15	1.3*E*6	2.5*E*5	16	15

^1^Concentrations of GlcN and GalN prepared for each experiment

^2^GlcN and GalN concentrations calculated using (3) and (4) with slope coefficients (*k*) derived from coefficients of slope from calibration curves determined from standard solutions analyzed within the same acquisition batch.

**Table 2:** Analyte-specific mass spectrometer parameters applied in the reported method: DP: declustering potential, FP: focusing potential, EP: entrance potential, CE: collision energy, and CXP: cell exit potential.

Analyte	Precursor ion (*m*/*z*)	Product ion (*m*/*z*)	DP (eV)	FP (eV)	EP (eV)	CE (eV)	CXP (eV)
Glucosamine/galactosamine	180 [M + H]^+^	72	25	100	12	23	13
Glucosamine/galactosamine	180 [M + H]^+^	84	25	100	12	21	13
Muramic acid	252 [M + H]^+^	126	30	90	9	25	13

**Table 3:** LC-MS/MS method performance of retention, linearity, sensitivity, precision, limit of detection, and limit of quantification for glucosamine, galactosamine, and muramic acid.

Analyte	*m*/*z* transition	*t*_*r*_ (min)	Linearity^a^	Sensitivity^b^	Prec. L/H^c^ (%)	LOD^d^ (*μ*M)	LOQ^e^ (*μ*M)
Glucosamine	180 > 72	4.41 ± 0.04	0.9997	3.8*E*4 ± 0.33*E*4	1.3/1.0	0.025	0.07
Glucosamine	180 > 84	4.41 ± 0.04	0.9997	2.1*E*4 ± 0.17*E*4	0.8/0.7	0.05	0.2
Galactosamine	180 > 72	4.52 ± 0.03	0.9999	8.1*E*4 ± 0.78*E*4	0.3/0.7	0.01	0.03
Galactosamine	180 > 84	4.52 ± 0.03	0.9997	3.8*E*3 ± 0.38*E*3	1.8^*∗*^/1.5	0.5	1.2
Muramic acid	252 > 126	2.01 ± 0.01	0.9994	5.1*E*4 ± 0.20*E*4	1.0/0.6	0.01	0.03

^a^Pearson correlation coefficient (*r*) determined for seven points, ranging from LOQ to 40 *μ*M (*n* = 6).

^b^Regression slope expressed in area units (counts)/*μ*M (*n* = 5).

^c^Precision expressed as relative standard deviation (RSD) for repeated injections at 1 *μ*M (L) and 10 *μ*M (H) (*n* = 8).

^d^Limit of detection reported as the injected concentration giving a peak height corresponding to three times the baseline noise level.

^e^Limit of quantification reported as the injected concentration giving a peak height corresponding to ten times the baseline noise level.

^*∗*^Precision at low level was investigated at a concentration of 2 *μ*M for the 180 > 84 transition of galactosamine, due to its relatively high LOD.
